# Quantitative and Qualitative Assessment of Enamel Surface Roughness Following High-Concentration Peroxide Bleaching: A Comparative In Vitro Study

**DOI:** 10.7759/cureus.83322

**Published:** 2025-05-01

**Authors:** Mamnoon Ghafir, Nida Mehmood, Leeza Bharati, Shreya Bhukal, Ritika Sethi, Aanchal Chaudhary, Seema Gupta

**Affiliations:** 1 Department of Conservative Dentistry and Endodontics, Institute of Dental Sciences, Bareilly, IND; 2 Department of Conservative Dentistry and Endodontics, Kothiwal Dental College and Research Centre, Moradabad, IND; 3 Department of Public Health Dentistry, All India Institute of Medical Sciences, Delhi, IND; 4 Department of Pedodontics and Preventive Dentistry, Adept Dental Clinics, Secunderabad, IND; 5 Department of Conservative Dentistry and Endodontics, Babu Banarasi Das College of Dental Sciences, Lucknow, IND; 6 Department of Orthodontics, Kothiwal Dental College and Research Centre, Moradabad, IND

**Keywords:** bleaching, carbamide peroxide, dental enamel, hydrogen peroxide, surface roughness

## Abstract

Introduction: Bleaching is a widely practiced aesthetic dental treatment, but high-concentration peroxide-based agents may negatively impact enamel integrity. This in vitro study aimed to compare enamel surface roughness after exposure to 35% hydrogen peroxide and 37% carbamide peroxide using both scanning electron microscopy (SEM) and contact profilometry. This study further explored the correlation between these two methods for evaluating surface topographical changes.

Materials and methods: Fifty enamel specimens were prepared from 30 extracted human premolars and randomly divided into three groups: Group 1 (control, n = 10), Group 2 (35% hydrogen peroxide, n = 20), and Group 3 (37% carbamide peroxide, n = 20). Bleaching was performed for 15 minutes per day for seven days. Surface roughness was assessed at baseline and after bleaching using a contact profilometer (Mitutoyo Surftest SJ-410, Mitutoyo Corporation, Kanagawa, Japan) and SEM (JEOL JSM-6510LV, JEOL Ltd., Tokyo, Japan). For profilometric analysis, the mean surface roughness (Ra) was calculated from three standardized points per specimen. SEM images at 1000× magnification were used to analyse three-dimensional topographic changes. Data were analysed using a mixed model analysis of variance (ANOVA), post-hoc Dunn-Bonferroni test, and Spearman correlation analysis, with significance set at p < 0.05.

Results: Both bleaching agents caused a significant increase in enamel surface roughness compared with the control group (p < 0.001). SEM detected more microstructural alterations than profilometry, as indicated by the strong method effect (effect size = 0.87, p = 0.001). Post-hoc comparisons showed that both bleaching agents significantly differed from the control, whereas no significant difference was found between them. Correlation analysis revealed high consistency between the SEM and profilometer measurements, particularly in the control and hydrogen peroxide groups.

Conclusion: Exposure to both 35% hydrogen peroxide and 37% carbamide peroxide significantly increased enamel surface roughness with comparable etching effects. SEM was found to be more sensitive than profilometry for detecting microstructural changes.

## Introduction

The need for an aesthetically pleasing smile has led to the widespread use of tooth whitening procedures in modern dentistry [1]. The most common agents used for this purpose are hydrogen peroxide and carbamide peroxide, both of which are employed in varying concentrations to achieve desirable whitening outcomes [2]. Although their effectiveness in lightening dental enamel is well documented, the implications of their use on enamel surface morphology remain a subject of ongoing investigation, particularly when these agents are used at high concentrations [3].

Hydrogen peroxide, typically in concentrations ranging from 10% to 40%, acts as a potent oxidizing agent that penetrates enamel and dentin to break down chromogenic compounds [4]. Carbamide peroxide, often used at concentrations of 10% to 37%, decomposes into hydrogen peroxide and urea, releasing approximately one-third of its weight as hydrogen peroxide in the process [5]. Although both agents share a similar bleaching mechanism, their interactions with the enamel surface differ owing to variations in pH, release rates, and exposure times.

From a clinical perspective, the priority is not only to achieve whitening but also to preserve the structural integrity of the enamel. Efficient, high-concentration bleaching agents raise concerns about potential morphological changes in enamel, such as increased porosity, surface roughness, and mineral loss [3]. These alterations, although sometimes subtle, can have lasting effects on enamel durability, resistance to wear, and susceptibility to staining and plaque accumulation.

Scanning electron microscopy (SEM) provides an extensive depth of field, remarkable spatial resolution, and rapid in situ measurements. SEM imaging allows for the visualization of the ultrastructure of enamel, capturing surface defects and textural variations that may not be detectable through traditional optical means [6]. When paired with surface profilometry or digital imaging software, it is possible to quantify the extent of surface alterations, thereby enabling a more comprehensive understanding of the effects of bleaching agents.

The rationale behind this study lies in the need to bridge the gap between aesthetic demands and enamel preservation. Although many in-office whitening systems utilize 35% hydrogen peroxide for immediate and dramatic results, at-home treatments may contain up to 37% carbamide peroxide, marketed as a gentler yet effective alternative [7]. However, comparative data on enamel surface changes induced by these two agents, particularly using a 3-Dimensional (3D) surface profile and SEM approach, remain limited.

This study aimed to investigate and compare 3D surface profile changes in enamel following exposure to 35% hydrogen peroxide and 37% carbamide peroxide. By employing SEM analysis, we sought to visualize and quantify the microscopic effects of these bleaching agents, thereby contributing to evidence-based recommendations for safer and more effective whitening practices. Understanding how these agents interact with enamel at a microstructural level will not only enhance clinical decision-making but also help guide patients toward informed choices about their oral health and cosmetic treatments.

## Materials and methods

Study design and setting

This in vitro, controlled, comparative study was conducted in the Department of Conservative Dentistry and Endodontics at the Kothiwal Dental College and Research Centre, Moradabad, India, from July 2024 to November 2024, following a standardized protocol for sample preparation, bleaching, and surface analysis. Ethical approval for this study was obtained from the Institutional Ethics Committee (KDCRC/IERB/05/2024/SH34). All teeth were collected following informed consent from patients undergoing routine orthodontic extractions, ensuring ethical compliance with the Declaration of Helsinki.

Sample size estimation

A minimum sample size of 48 was estimated at 80% power and 5% alpha error using an effect size of 0.46 for surface roughness between carbamide peroxide and hydrogen peroxide from a previous study [7]. G*Power software version 3.6.9 (Heinrich-Heine-Universität Düsseldorf, Germany) was used for the sample size estimation.

Sample selection and preparation

Thirty freshly extracted human premolars, free from caries, cracks, restorations, fluorosis, structural defects, staining, and enamel hypoplasia, were selected for the study. Following extraction, the teeth were cleaned for soft tissue debris using an ultrasonic scaler (Woodpecker, Guilin Woodpecker Medical Instrument Co., Guilin, China) and stored in distilled water at 4^0^C to maintain hydration until use.

**Figure 1:**
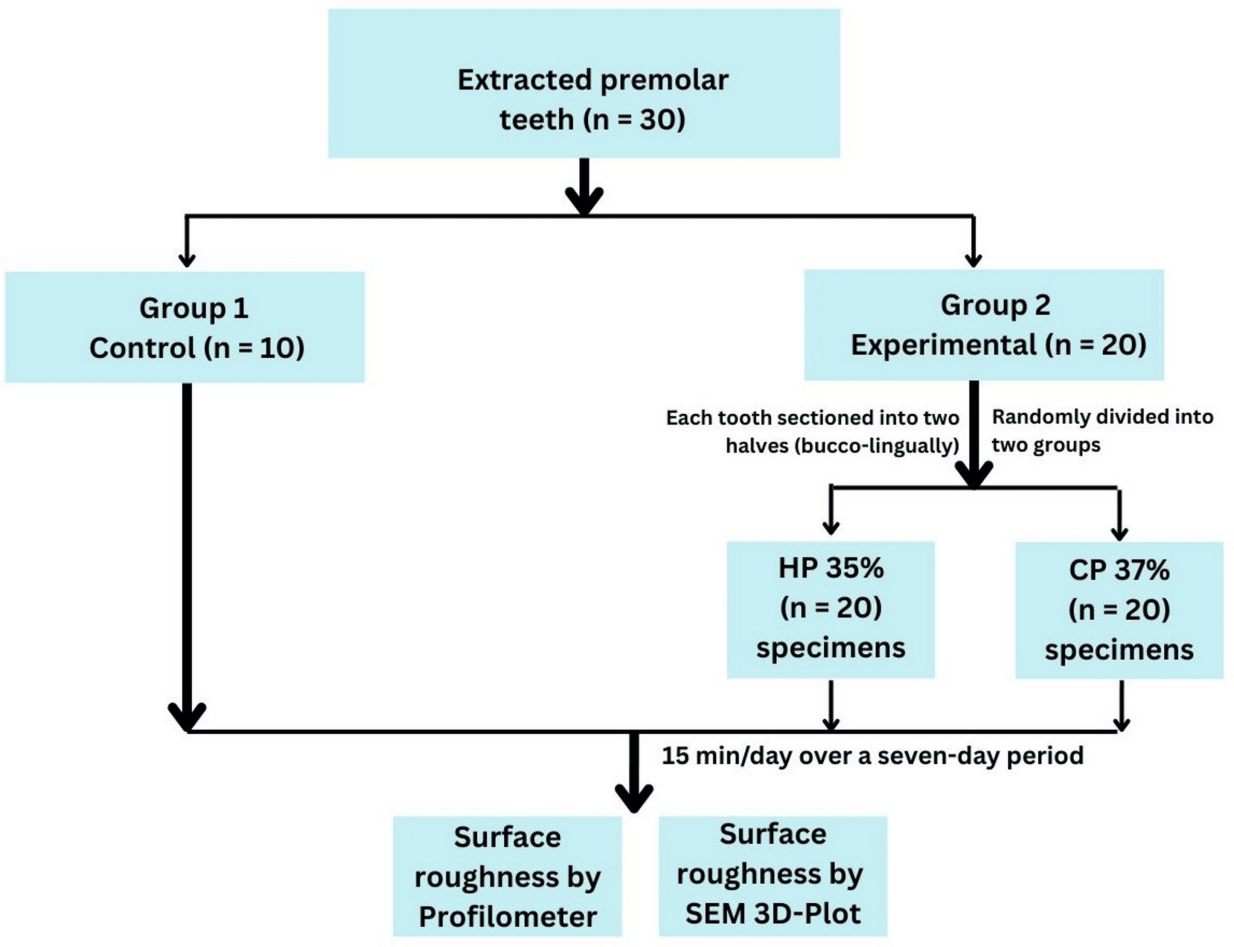
Study design. HP: hydrogen peroxide; CP: carbamide peroxide; SEM: scanning electron microscopy, 3D: 3-Dimensional.

To standardize the sample dimensions and optimize specimen utilization, 20 selected premolars were sectioned longitudinally in the buccolingual direction using a diamond disc (Horico, Berlin, Germany) under copious water cooling. This sectioning approach was specifically chosen to prevent potential bias arising from variations in enamel mineral content between teeth from different patients. By using both halves of the same tooth across the experimental groups, intra-tooth consistency was maintained, thereby minimizing inter-individual variability and enhancing the internal validity of the study. This process yielded 40 enamel specimens, which were randomly allocated to two experimental groups (n = 20 per group). The remaining 10 intact premolars served as the control group (n = 10). An allocation ratio of 2:2:1 (experimental: experimental: control) was selected to ensure a sufficient sample size for intergroup comparison, while preserving an appropriate control baseline for evaluating untreated enamel. A total of 50 enamel specimens from 30 extracted premolar teeth were included in the final analysis (Figure 1).

Each specimen was separately placed in a self-curing acrylic resin within a ring apparatus, and the enamel surfaces were meticulously abraded using 600- and 1200-grit silicon carbide paper with the application of water cooling to establish a uniform buccal enamel surface.

Methodology

Fifty enamel specimens were divided into three groups as follows: Group 1 (control) (n = 10), where no bleaching treatment was done, Group 2 (n = 20) was treated with 35% hydrogen peroxide gel, and Group 3 (n = 20) was treated with 37% carbamide peroxide gel. Two commercially available bleaching agents were used: 35% hydrogen peroxide gel (Opalescence Boost, Ultradent Products Inc., South Jordan, UT, USA) and 37% carbamide peroxide gel (Opalescence PF 37%, Ultradent Products Inc.). These concentrations were selected based on their prevalent clinical use in both in-office and high-strength at-home whitening systems. The 35% hydrogen peroxide formulation allows for rapid bleaching, whereas the 37% carbamide peroxide serves as a slower-release yet high-potency alternative. This comparison aimed to simulate real-world exposure levels and evaluate their respective effects on enamel morphology [8].

In Group 2, the gel was applied directly onto the enamel surface using a microbrush in a uniform 1-2 mm layer. Each application session lasted 15 minutes/day over a seven-day period, which aligned with the manufacturer’s recommendations for in-office procedures. After each session, the specimens were thoroughly rinsed with distilled water for 30 s to remove residual gel and then gently dried with absorbent paper.

In Group 3, the gel was also applied in a uniform layer using a microbrush. Although carbamide peroxide is typically used for extended durations in at-home settings, to allow for a standardized comparison with hydrogen peroxide, each specimen in this group was also treated for 15 minutes/day over a seven-day period. This controlled protocol ensured equivalence in exposure time and minimized potential variability due to different treatment durations. The specimens were rinsed and dried after each application.

Each bleaching session lasted 15 minutes and consisted of three 5-minute gel applications per specimen. The bleaching gel was renewed every 5 minutes to maintain consistent peroxide activity and to ensure uniform exposure across the enamel surface. This renewal protocol aligns with the manufacturer’s guidelines and mimics clinical practice, particularly for hydrogen peroxide, which is known to lose effectiveness due to rapid breakdown upon contact with tooth surfaces and ambient conditions. After each session, all the specimens were thoroughly rinsed with distilled water for 30 s and dried with absorbent paper.

All treatments were conducted at room temperature in a dry, clean environment, with the samples placed on an acrylic tray during gel application to avoid cross-contamination. Between sessions, the specimens were stored in artificial saliva (Wet Mouth, ICPA Health Products Ltd., Mumbai, India) at 37^0^C to replicate oral conditions and maintain enamel hydration. Surface analysis was performed at baseline (T0) and after one week of treatment (T1). All tooth shade measurements were performed by an experienced examiner who was blinded to the bleaching agent used and its concentration to minimize bias and ensure consistency (RS and NM).

Surface profilometry analysis

Surface roughness was assessed using a contact profilometer (Mitutoyo Surftest SJ-410, Mitutoyo Corporation, Kanagawa, Japan). Measurements were taken from three predefined points on each enamel specimen and the mean surface roughness value (Ra, in micrometers) was calculated. All readings were performed under standardized conditions, with careful positioning of the samples to ensure reproducibility.

SEM analysis

**Figure 2:**
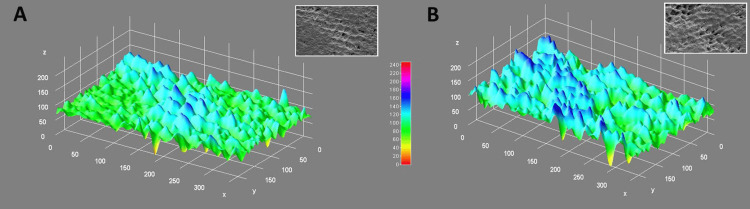
A) 3-Dimensional (3D) surface plot analysis of enamel with 37% carbamide peroxide, B) 3D surface plot analysis of enamel with 35% hydrogen peroxide. This figure depicts data of samples included in this study. X axis denotes 0-300 gray value, Y axis denotes 0-150 gray value, Z axis denotes 0-200 gray value.

Following profilometry, enamel surface morphology was evaluated using SEM. Samples were dehydrated in ascending concentrations of ethanol, mounted on aluminium stubs, sputter-coated with gold using a sputtering unit (Quorum Q150R Plus, Quorum Technologies, East Sussex, UK), and examined under a SEM (JEOL JSM-6510LV, JEOL Ltd., Tokyo, Japan) at 1000× magnification. Representative micrographs were captured for each specimen to document the surface alterations (Figure 2).

Calibration and reliability testing

Prior to data collection, both the profilometer and the SEM equipment were calibrated using standard reference materials in accordance with the manufacturer’s protocols. Surface profilometry and SEM analyses were performed independently by two trained and blinded examiners (MG, SB) who were affiliated with a different institution from where the study was conducted. This measure was intentionally taken to minimize potential observational bias and ensure objective interpretation of the results. By involving external evaluators who were unaware of the group allocation and bleaching protocols, the reliability and impartiality of the data analysis were further strengthened. Inter-examiner reliability was calculated using the intraclass correlation coefficient (ICC), yielding values of 0.92 for profilometry and 0.89 for SEM, both indicating excellent agreement. Any discrepancies in SEM interpretations were resolved through consensus.

Statistical analysis

The data was analysed by a statistician (AC) who was provided with coded data to eliminate bias in analysis. The data were systematically analysed using SPSS software version 23.0 (IBM Corp., Armonk, NY, USA). The study’s surface roughness data underwent a normality assessment via the Shapiro-Wilk test, revealing a non-normal distribution, verified by a Q-Q plot. Mean surface roughness values derived from profilometer measurements and 3D surface analysis of SEM images were compared across study groups using the mixed-model analysis of variance (ANOVA), followed by post-hoc analysis by Dunn-Bonferroni test. Additionally, Spearman correlation analysis was employed to evaluate the relationship between the surface roughness assessed using these two methods. The level of significance was set at p < 0.05.

## Results

The analysis demonstrated a clear trend of increased surface roughness in enamel samples treated with 37% carbamide peroxide and 35% hydrogen peroxide compared with the control. Both the 3D SEM surface plots and profilometer Ra measurements consistently indicated elevated roughness in the experimental groups, supporting the hypothesis that high-concentration bleaching agents induce significant surface alterations. The 3D SEM data also suggest a higher degree of variability, likely reflecting greater sensitivity to microstructural changes, whereas the profilometer provides more consistent quantification. Overall, these results confirm that peroxide-based agents contribute to enamel surface degradation through chemical etching. Although both experimental groups showed greater surface roughness than the control, 37% carbamide peroxide produced less surface roughness than 35% hydrogen peroxide (Table 1). 

**Table 1:** Descriptive analysis of surface roughness analyzed by Profilometer and 3D surface plot of SEM images post-bleaching. CP: carbamide peroxide, HP: hydrogen peroxide, 3D: 3-Dimensional, SEM: scanning electron microscopy at 1000x magnification, CI: confidence interval. Data is presented in form of mean ± standard deviation (SD).

Outcome measures	Groups	Minimum	Maximum	95% CI for mean	Mean ± SD
Surface roughness 3D surface plot (SEM) (gray value)	Control	78	178	117.76 - 147.44	132.6 ± 31.71
	CP 37%	85	245	161.59 - 203.21	182.4 ± 44.46
	HP 35%	98	228	165.92 - 203.88	184.9 ± 40.54
Surface roughness (Ra) Profilometer (micrometer)	Control	0.15	0.26	0.19 - 0.23	0.19 ± 0.04
	CP 37%	0.16	0.28	0.22 - 0.26	0.23 ± 0.26
	HP 35%	0.16	0.29	0.24 - 0.28	0.26 ± 0.04

**Figure 3:**
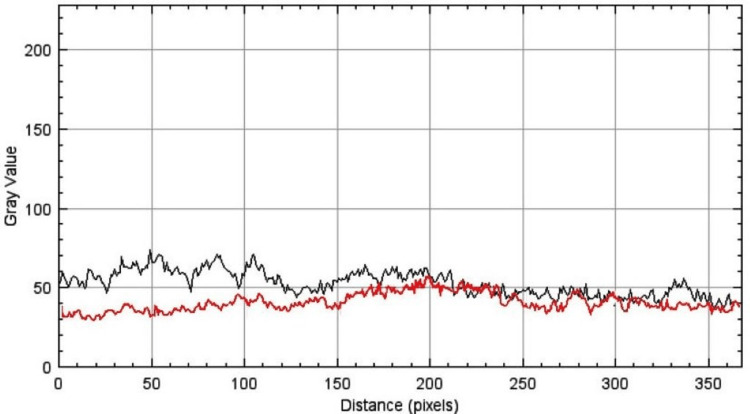
The surface plot profile of enamel etched by 35% hydrogen peroxide (black wave) and 37% carbamide peroxide (red wave). This figure was based on data of the study.

The mixed-model ANOVA revealed that both the method of measurement and the treatment group had significant effects on surface roughness. The strong effect size (0.87) and low p-value (0.001) for the comparison between SEM and the profilometer indicate that the method used substantially influenced the roughness values, with SEM being more sensitive in detecting surface changes (Figure 3).

Additionally, the significant interaction between the repeated measures factor and group (p = 0.001, effect size = 0.02) suggested that the impact of bleaching agents on surface roughness differed depending on the method used. This implies a group-specific response, where enamel surfaces treated with peroxide agents exhibited more pronounced roughness when evaluated by SEM, highlighting their greater sensitivity to etching-induced microstructural alterations (Table 2).

**Table 2:** Mixed model analysis of variance (ANOVA) for outcomes variables (surface roughness by SEM and profilometer). RM factor: surface roughness, SEM: scanning electron microscopy at 1000x magnification, *p-value < 0.05: significant.

Variables	Sum of squares	df	Mean square	F stat	p-value	Effect size
Surface roughness (SEM), and Profilometer	830578.95	1	830578.95	1079.27	0.001*	0.87
Groups	17438.27	2	8719.13	11.29	0.001*	0.02
RM Factor x Groups	17372.3	2	8686.15	11.29	0.001*	0.02

Post-hoc surface profilometric analysis using the Dunn-Bonferroni test confirmed that both 37% carbamide peroxide and 35% hydrogen peroxide significantly increased enamel surface roughness compared to the control group. The adjusted p-values (CP: 0.002, HP: 0.001) support the presence of statistically significant differences, whereas the absence of a significant difference between the carbamide peroxide and hydrogen peroxide groups indicates that both agents exert a similar etching effect on enamel. These results highlight the comparable impact of high-concentration peroxide bleaching agents on enamel integrity and underscore the need for careful material selection in clinical settings to minimize surface damage (Table 3).

**Table 3:** Post-hoc analysis with Dunn-Bonferroni test for profilometer method. Adjusted p-value: value adjusted with Bonferroni correction, CP: carbamide peroxide, HP: hydrogen peroxide, *p-value < 0.05: significant.

Profilometer	Test Statistic	Standard Error	Standard Test Statistic	p-value	Adjusted p-value
Control - CP 37%	-19.2	5.52	-3.48	0.001*	0.002*
Control - HP 35%	-21.6	5.52	-3.92	0.001*	0.001*
CP 37% - HP 35%	-2.4	5.52	-0.44	0.663	1

Post-hoc analysis of 3D surface plot (SEM) roughness confirmed that both 37% carbamide peroxide and 35% hydrogen peroxide significantly increased enamel surface roughness compared to the control group, as indicated by the adjusted p-values of 0.001 for both comparisons. Similar test statistics and standard errors for carbamide peroxide and hydrogen peroxide suggested that these agents exert comparable etching effects on enamel surfaces. These results were consistent with profilometer findings, reinforcing the conclusion that high-concentration peroxide treatments produced a significant and comparable increase in enamel roughness. This consistency across measurement methods underscores the reliability of the observed effects and highlights the need for caution when selecting bleaching agents to preserve enamel integrity in clinical practice (Table 4).

**Table 4:** Post-hoc analysis with Dunn-Bonferroni test for 3D surface plot method by SEM. Adjusted p-value: value adjusted with Bonferroni correction, CP: carbamide peroxide, HP: hydrogen peroxide, SEM: scanning electron microscopy at 1000x magnification, *p-value < 0.05: significant.

SEM 3D surface plot	Test Statistic	Standard Error	Standard Test Statistic	p-value	Adjusted p-value
Control - CP 35%	-19.2	5.46	-3.52	0.001*	0.001*
Control - HP 35%	-20.4	5.46	-3.74	0.001*	0.001*
CP 35% - HP 35%	-1.2	5.46	-0.22	0.826	1

Spearman correlation analysis demonstrated a statistically significant and highly positive relationship between the surface roughness measurements obtained by SEM and profilometry across all groups. A perfect correlation was observed in the control group, whereas strong correlations were noted in both the 37% carbamide peroxide and 35% hydrogen peroxide groups. The overall correlation was exceptionally high, indicating strong consistency between the two measurement methods. These findings suggest that SEM and profilometer are reliably aligned in detecting enamel surface changes, particularly in untreated and hydrogen peroxide-treated samples, supporting their interchangeable use for assessing the effects of bleaching agents on enamel roughness (Table 5).

**Table 5:** Correlation between both methods by Spearman test. *p-value < 0.05: significant, SEM: scanning electron microscope at 1000x, Very Strong: 0.8≤∣r∣≤1.

Outcome variables	Groups	r value	p-value
Surface roughness (SEM) and Surface roughness Profilometer	Control	1	0.001*
	CP 37%	0.88	0.001*
	HP 35%	0.97	0.001*
	Total	0.98	0.001*

## Discussion

This study investigated the effects of two high-concentration bleaching agents, 37% carbamide peroxide and 35% hydrogen peroxide, on enamel surface roughness using two complementary assessment methods: 3D surface plots from SEM and profilometric measurements. The findings revealed that both bleaching agents significantly increased enamel surface roughness compared with the untreated control group, with minor differences observed between the two agents in terms of severity.

The elevation in surface roughness observed post-bleaching supports the hypothesis that high-concentration peroxide-based agents induce chemical etching of the enamel, a process that degrades the enamel matrix and increases its irregularity. These results are consistent with those of several previous studies that demonstrated similar enamel alterations following peroxide exposure. For instance, Bitter [9] and Sa et al. [10] observed a roughened enamel surface after the application of bleaching agents, attributing the changes to the loss of mineral content and disruption of the organic matrix. Moreover, Owda et al. [11] found that both carbamide peroxide and hydrogen peroxide bleaching agents resulted in increased surface roughness, with SEM images revealing pitting and erosion in the bleached enamel. In an SEM analysis, Haywood et al. [12] observed a lack of morphological alterations in the enamel surface following administration of a 10% carbamide peroxide bleaching agent. Furthermore, Ernst et al. [13] documented negligible, inconsequential, or absent modifications on enamel surfaces when examined at a magnification of 3000x while utilizing 30% solutions of hydrogen peroxide.

In our study, SEM-based 3D surface plots showed greater absolute roughness values and more variability than profilometer readings, indicating the higher sensitivity of SEM in capturing microstructural changes. This is consistent with the findings of Fuzzi et al. [14], who emphasized that SEM enables the detection of microetching patterns that are not evident in profilometric data. The increased sensitivity of the SEM likely explains the more pronounced differences among the groups when this method was employed.

Interestingly, although both agents significantly increased surface roughness, 35% hydrogen peroxide showed marginally higher roughness values than 37% carbamide peroxide. This may be attributed to the more immediate and aggressive oxidative action of hydrogen peroxide, which releases a higher concentration of reactive oxygen species (ROS) in a shorter time than carbamide peroxide, which decomposes more slowly and delivers a sustained release of peroxide [15,16]. It is probable that the 37% carbamide peroxide employed in the investigation yielded approximately 12% hydrogen peroxide, thereby engendering a comparatively lower production of ROS, hyperalgesia, and inflammatory mediators than the 35% hydrogen peroxide [8]. Peixoto et al. [8] reported less tooth sensitivity with 37% carbamide peroxide than with 35% hydrogen peroxide. Farawati et al. [17] found no surface alterations with 10%, 35%, or 44% carbamide peroxide. This disparity in the kinetics of active oxygen liberation necessitates an increased number of treatment sessions with carbamide peroxide to achieve outcomes analogous to those derived from hydrogen peroxide. Monteiro et al. [18] demonstrated that the application of 37% carbamide peroxide over three consecutive days, administered bi-daily, yielded bleaching efficacy comparable to that of 35% hydrogen peroxide utilized across three distinct bleaching sessions with a one-week interval, thereby suggesting that it is a viable alternative for achieving expedited results.

These differences in degradation kinetics could influence the extent of enamel alteration, although our statistical findings suggest that the differences between the bleaching agents were not significant. This corroborates the findings of Monteiro et al. [18] and de Boa et al. [19], who reported no substantial differences in enamel surface properties between similar concentrations of hydrogen peroxide and carbamide peroxide.

The mixed model ANOVA results further confirmed that both the bleaching agents and measurement method significantly affected the surface roughness outcomes. The large effect size for method comparison (0.87) underscores the importance of considering the inherent sensitivity and resolution of different assessment techniques when evaluating surface changes. The significant interaction between group and repeated measures indicated that the extent of roughness alteration depended not only on the bleaching agent, but also on the method used for quantification. This emphasizes the need for standardized protocols in future research to ensure consistency and comparability of data across studies.

Both the carbamide peroxide and hydrogen peroxide groups showed statistically significant increases in surface roughness compared to the control, reaffirming their etching effect on enamel. The absence of significant differences between the agents across both methods suggests that the choice of bleaching agent, when used at these concentrations, may not substantially influence the degree of surface alteration. These findings align with those of de Boa et al. [19], who reported similar enamel effects for both agents, particularly when high concentrations were used.

Clinical implications

The results of the present study have several clinical implications. First, the significant increase in enamel roughness following exposure to both 37% carbamide peroxide and 35% hydrogen peroxide highlights the potential for structural enamel degradation during in-office bleaching. Increased roughness can make enamel more susceptible to staining, plaque accumulation, and potential mechanical wear, thereby compromising long-term dental health and aesthetics. Second, the similarity in roughness outcomes between both bleaching agents suggests that both agents, when used at high concentrations, pose a comparable risk to enamel integrity. This underscores the importance of cautious application, patient-specific risk assessment, and post-bleaching remineralization strategies. Dentists may consider incorporating remineralizing agents, such as fluoride, calcium phosphate, or casein phosphopeptide-amorphous calcium phosphate (CPP-ACP), following bleaching to aid enamel recovery [20].

Limitations

Despite the strengths of this study, it has several limitations that must be acknowledged. First, the color change was not evaluated along with the surface roughness. As bleaching efficacy is typically measured by shade improvement, future studies should correlate color changes with surface integrity to establish a more holistic understanding of the trade-offs involved. Second, although in vitro sensitivity testing was conducted, it may not fully replicate the in vivo conditions of the oral cavity, such as the presence of saliva, temperature fluctuations, and mechanical forces, such as mastication. These factors may influence the actual extent of enamel damage and patient discomfort. Another limitation is the exclusive focus on surface roughness without evaluating mineral content changes or the mechanical properties of the enamel post-treatment. Techniques such as energy-dispersive X-ray spectroscopy (EDX) and nanoindentation can offer additional insights into compositional and structural changes. Lastly, the study did not assess the long-term outcomes or the effects of multiple bleaching sessions. As many patients undergo repeated whitening procedures, future investigations should consider the cumulative effects of bleaching on enamel and the role of remineralization protocols.

## Conclusions

Within the limitations of this in vitro study, it can be concluded that both 37% carbamide peroxide and 35% hydrogen peroxide significantly increased enamel surface roughness compared with the untreated control. Although both agents demonstrated comparable etching effects, 35% hydrogen peroxide produced slightly higher roughness values. SEM revealed greater sensitivity in detecting microstructural changes than profilometry, although both methods showed strong agreement and reliability. These findings suggest that high-concentration bleaching agents can compromise enamel surface integrity, emphasizing the need for careful clinical use and consideration of post-bleaching remineralization strategies to preserve enamel health.
